# Comparison of postoperative outcomes of mini percutaneous nephrolithotomy and standard percutaneous nephrolithotomy: a meta-analysis

**DOI:** 10.1007/s00240-022-01349-8

**Published:** 2022-08-11

**Authors:** Chuanping Wan, Daoqi Wang, Jiajia Xiang, Bin Yang, Jinming Xu, Guiming Zhou, Yuan Zhou, Yuan Zhao, Jiao Zhong, Jianhe Liu

**Affiliations:** 1 Department of Urology, The Second Affiliated Hospital of Kunming Medical University, 374 Dianmian Avenue, Wuhua District, Kunming, 650101 NO China; 2 Department of Anesthesiology, 920Th Hospital of Joint Logistics Support Force, PLA, Kunming, Yunnan People’s Republic of China

**Keywords:** Kidney stones, Mini-PCNL, Standard-PCNL, Tract size, Stone-free rate, Complications

## Abstract

Our study was aimed to evaluate the postoperative outcomes of Mini Percutaneous Nephrolithotomy (Mini-PCNL) and Standard Percutaneous Nephrolithotomy (Standard-PCNL) to determine the optimum option for patients with renal calculi. For publications published between January 2010 and April 2021, a comprehensive search of the PubMed, Cochrane Library, Web of Science, and EMBASE databases was done. The literatures were chosen based on the criteria for inclusion and exclusion. After the data were retrieved and the quality was assessed, the meta-analysis was performed using Review Manager Software (RevMan 5.4.1, Cochrane Collaboration, Oxford, UK). We selected 20 trials with a total of 4953 people out of 322 studies. There were 2567 patients treated with Mini-PCNL and 2386 patients treated with Standard-PCNL. Meta-analysis results showed no difference in stone-free rates (SFR, *P* = 0.93), fever (*P* = 0.83), and postoperative pain (VAS score) (*P* = 0.21) between Mini-PCNL and Standard-PCNL. Patients in the Mini-PCNL group experienced shorter hospital stay (*P* < 0.0001), less hemoglobin drop (*P* < 0.00001), less blood transfusion (*P* < 0.00001), higher postoperative tubeless (*P* = 0.0002), and fewer complications including bleeding (*P* = 0.01), perforation (*P* = 0.03), and leakage (*P* = 0.01). Compared with Standard-PCNL, operative time was longer in the Mini-PCNL group (*P* = 0.0005). Mini-PCNL had a shorter hospital stay, less hemoglobin drop, less blood transfusion, greater postoperative tubeless, fewer complications, and a longer operational time when compared to Standard-PCNL. SFR, fever, and postoperative pain were similar in both of them. Mini-PCNL may be a superior option for patients with proper size renal calculi.

**Supplementary Information:**

The online version contains supplementary material available at 10.1007/s00240-022-01349-8.

## Introduction

The surgical standard for treating large or difficult kidney stones is percutaneous nephrolithotomy (PCNL) [1]. With the maturation of technology and the advancement of medical expertise, smaller sheaths have become increasingly used for PCNL during the last two decades. Despite the lack of a globally accepted word for PCNL tract size, procedures with an outer sheath greater than 24 Fr are considered standard PCNL procedures [2]. Mini-PCNL is defined by the European Association of Urology (EAU) as a tract size of less than 22 Fr. [3]. At present, there are many comparisons between Mini-PCNL and Standard-PCNL, but there are some controversies, so we compared the SFR, operation time, hospital stay, hemoglobin drop, blood transfusion, postoperative pain (VAS score), postoperative tubeless, and complications of Mini-PCNL and Standard-PCNL over the last decade in the hopes of obtaining an evidence-based basis that would assist clinicians in choosing surgical options.

## Materials and methods

### Search strategy

The meta-analysis was carried out by looking for publications published between January 2010 and April 2021 in the PubMed, Cochrane Library, Web of Science, and EMBASE databases. The search details were: ((("Kidney Calculi"[Mesh]) OR ((((((((((((Calculi, Kidney) OR (Calculus, Kidney)) OR (Kidney Calculus)) OR (Nephrolith)) OR (Renal Calculus)) OR (Kidney Stones)) OR (Kidney Stone)) OR (Stone, Kidney)) OR (Stones, Kidney)) OR (Renal Calculi)) OR (Calculi, Renal)) OR (Calculus, Renal))) AND (("Nephrolithotomy, Percutaneous"[Mesh]) OR (((Nephrolithotomies, Percutaneous) OR (Percutaneous Nephrolithotomies)) OR (Percutaneous Nephrolithotomy)))) AND (mini). The search was limited to publications in English.

### Inclusion and exclusion criteria

Before beginning the literature search, inclusion and exclusion criteria were established. The studies mentioned met the following criteria: (a) comparison of Mini-PCNL and Standard-PCNL; (b) at least one of our interesting data (including basic characteristics (Table1), surgical procedures, SFR, operation time, length of hospital stay, hemoglobin drop, blood transfusions, postoperative pain, tubeless PCNL rate, and complications) is found in the literature. Exclusion criteria included: (a) Incomplete analytical data; (b) Pediatric patients under 18 years of age; (c) Super Mini-PCNL (12-14F), Ultra mini-PCNL (10-13F), Micro-PCNL (4F)[3]; (c) Data cannot be extracted.

**Table 1:** Characteristics of included studies

study	Design	Procedures	Sample size	Age (year)	Sex (M/F)	side (R/L)	BMI, kg/m2	Stone Size, mm
Bozzini, G. 2020	RCT	Mini-PCNL Standard-PCNL	47 44	55.8 53.3	20/27 23/21	22/25 25/19		16.82 16.38
Cheng,F. 2010	RCT	Mini-PCNL Standard-PCNL	72 115	37.2 39.6	39/33 63/52	43/29 67/48		
Du, C. 2018	RCT	Mini-PCNL Standard-PCNL	304 297	41.2 ± 16.9 44.5 ± 18.7	181/123 179/118	147/157 151/146		
Guler,A. 2019	RCT	Mini-PCNL Standard-PCNL	51 46	46.9 ± 13.7 47.4 ± 13.9	29/22 23/23	29/22 25/21	28.5 ± 5.6 29.6 ± 5.9	38.7 ± 13.1 42.8 ± 22.5
Kandemir,E. 2020	RCT	Mini-PCNL Standard-PCNL	76 72	47.0 ± 13.9 46.7 ± 14.2	50/26 48/24	40/36 35/47	28.6 ± 5.4 28.4 ± 5.6	32.6 ± 8.1 33.1 ± 10.9
Kukreja,R. A. 2018	RCT	Mini-PCNL Standard-PCNL	61 62	41.95 ± 13.53 40.3 ± 14.2		33/28 30/32	27.1 ± 5.87 25.54 ± 3.58	20.6 ± 3.47 21.5 ± 3.53
Sakr, A. 2017	RCT	Mini-PCNL Standard-PCNL	75 75	43.8 40.2	40/35 52/23	51/36 33/48	28.4 27.8	27 26
Tepeler, A. 2014	RCT	Mini-PCNL Standard-PCNL	10 10	47.2 44.3	4/6 6/4		27.5 27.8	19.9 21.9
Thakur, A. 2021	RCT	Mini-PCNL Standard-PCNL	30 30	34.5 ± 16.32 32.4 ± 12.6	21/9 17/13		26.32 ± 5.10 25 ± 5.16	17.9 ± 5 19.4 ± 5.3
Zeng, G. 2021	RCT	Mini-PCNL Standard-PCNL	992 988	51 51	526/466 531/457	500/492 487/501	24.4 24.7	29 29
Zhong,W. 2011	RCT	Mini-PCNL Standard-PCNL	29 25	41 38	14/15 11/14			
Abdelhafez, M. F. 2016	Non-RCT	Mini-PCNL Standard-PCNL	71 62	52 58	37/34 31/31	29/42 21/41	26.2 26.4	38.6 38.2
ElSheemy,M.S. 2019	Non-RCT	Mini-PCNL Standard-PCNL	378 151	37.08 ± 12.62 43.42 ± 13.21	137/241 58/93	206/172 75/76	27.2 ± 2.22 27.03 ± 2.16	
Hamamoto, S. 2014	Non-RCT	Mini-PCNL Standard-PCNL	19 82	48.9 53.2	12/7 66/16	5/14 22/60	24.8 24.6	
Khadgi, S. 2021	Non-RCT	Mini-PCNL Standard-PCNL	83 70	43.7 ± 13.9 51.9 ± 9.7	44/39 32/38	36/47 21/41	29 ± 3.3 34 ± 6	
Knoll,T. 2010	Non-RCT	Mini-PCNL Standard-PCNL	25 25	52 ± 11.6 48 ± 15.5	16/9 17/8		27 ± 3.5 29 ± 5.6	18 ± 3.3 22 ± 4.25
Li,L.Y. 2010	Non-RCT	Mini-PCNL Standard-PCNL	93 72	51.5 49.2	56/37 43/29	48/45 31/41		28.6 30.4
Mishra,S. 2011	Non-RCT	Mini-PCNL Standard-PCNL	26 26	42.2 ± 19.8 48.2 ± 16.8	18/8 18/8	8/19 10/18	23.8 ± 2.6 22.6 ± 2.7	
Sabnis, R. B. 2020	Non-RCT	Mini-PCNL Standard-PCNL	11 20	40.2 ± 15.1 49.2 ± 11.5	5/6 16/4			
Wu, C. 2017	Non-RCT	Mini-PCNL Standard-PCNL	114 114	47.6 ± 8.2 48.1 ± 7.9	69/45 68/46	59/55 55/59	23.0 ± 2.7 22.8 ± 2.8	34 ± 10 33 ± 11

### Data extraction

In this paper, the primary outcomes studied were SFR, operative time, length of hospital stay, hemoglobin drop, blood transfusion, postoperative pain (VAS score), postoperative tubeless and related complications. We collected the author's name, publication period, study type, sample size, average age of patients, gender ratio, stone location, stone size, SFR, operation time, hospital stay, hemoglobin drop, blood transfusion, postoperative pain (VAS Score), postoperative tubeless and related complications from the final included literature. The complications include: fever, bleeding, renal pelvis perforation, urine leakage. For identifying purposes, the first author's name and the year the piece was published were utilized. Two reviewers separately extracted data and came to an agreement on all issues.

### Assessment of study quality

The Oxford Centre for Evidence-Based Medicine provides criteria for grading the level of evidence (LE) for each included study. The Jadad scale [4] for randomized controlled trials (RCTs) (Supplementary Table 1) and the Newcastle–Ottawa Scale (NOS) for non-randomized controlled trials (Non-RCTs) (Supplementary Table 2,3) were used to assess the methodological quality of the investigations. The full texts of the included literatures were read and independently assessed by two researchers. If the assessment results of two researchers were inconsistent, the third person performed re-assessment.

### Statistical analysis

For statistical analysis, we utilized RevMan 5.4.1 software from the Cochrane Collaboration. The summary statistic for dichotomous variables was the Pooled Risk Ratio (RR). For continuous variables, the mean difference (MD) was determined. The 95% confidence interval (CI) for both RR and MD was provided, and *P* < 0.05 was considered statistically significant. *I*^2^ statistics were used to assess the studies' heterogeneity [5]. The fixed-effect model was used if the heterogeneity was less than 50%, else the random-effects model was used [6]. If there is heterogeneity among the study results (*I*^2^ ≥ 50%), the causes of heterogeneity were analyzed one by one study until gaining the best homogeneity.

## Results

### Study selection and characteristics

**Fig.1:**
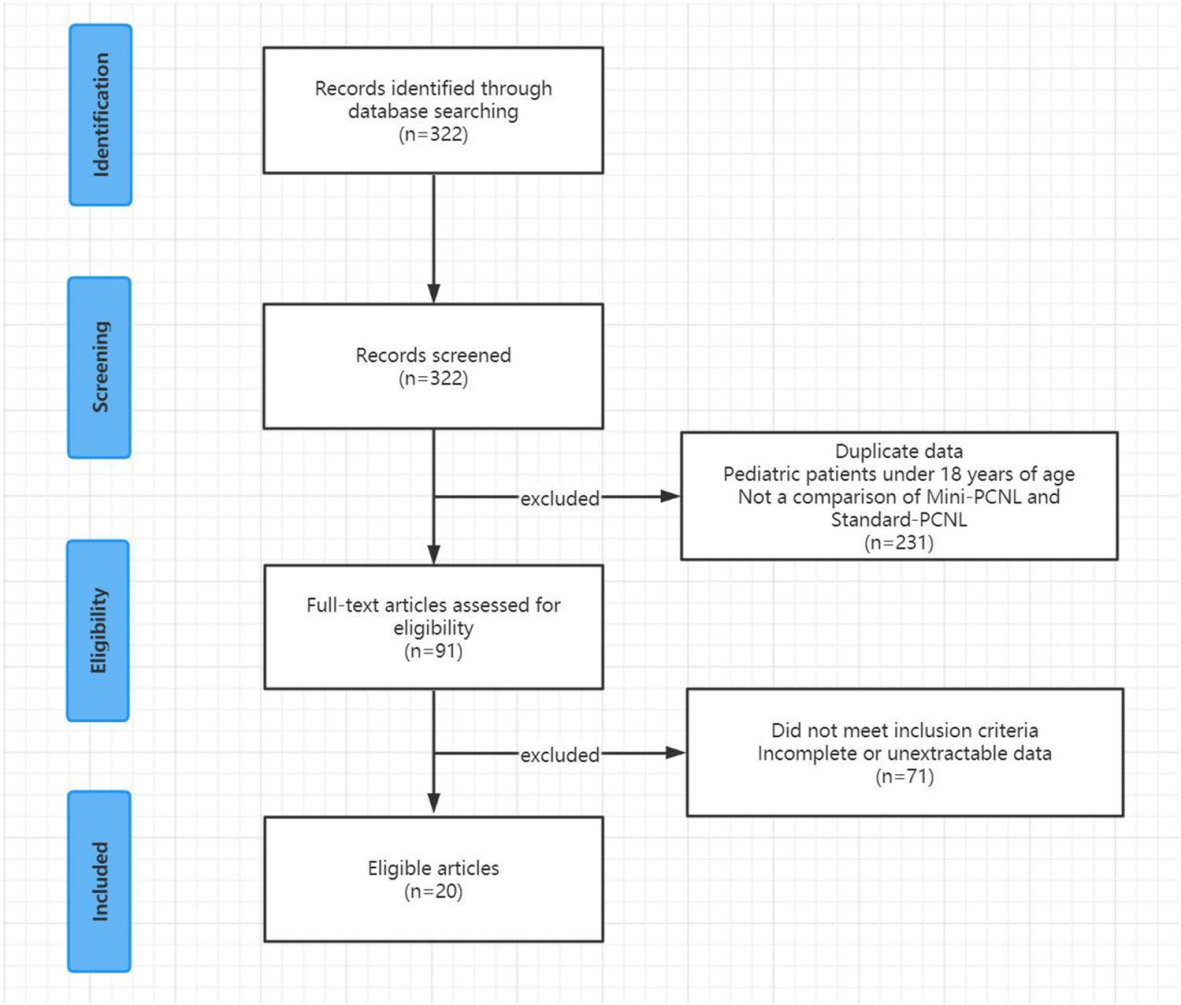
Study details flow chart

The literature search resulted in the discovery of 322 potentially relevant publications. After removing 231 irrelevant articles, 91 items were further evaluated. Finally, our meta-analysis included 20 publications. (Fig. 1)[7–26]. A total of 4953 participants were included in the study, with 2567 receiving Mini-PCNL and 2386 receiving Standard-PCNL. This research includes 11 RCTs [8–10, 12, 14, 17, 21–23, 25, 26] and 9 non-RCTs [7, 11, 13, 15, 16, 18–20, 24]. Table 1 shows the main characteristics of the studies that were considered.

### SFR and subgroup analysis

**Fig.2:**
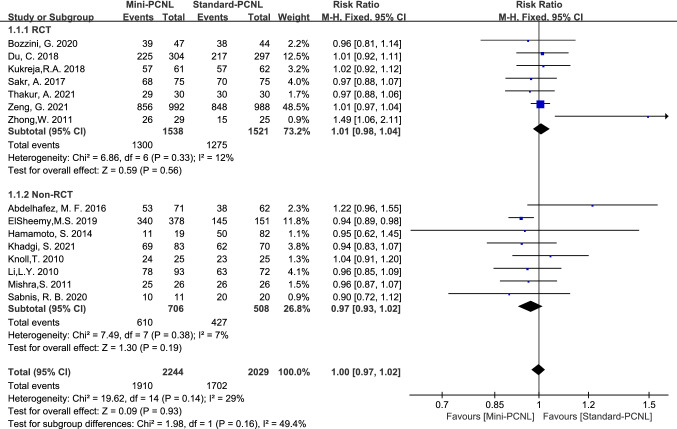
Forest plot for SFR

15 studies reported SFR, with good homogeneity among the studies (*P*=0.15, *I*^2^=29%). SFR was 85.1% (1910 of 2244 patients) of Mini-PCNL and 83.9% (1702 of 2029 patients) of Standard-PCNL with no significant difference (Risk Ratio (RR) =1.00, 95%Confidence Interval (CI) 0.97–1.02, P=0.93; Fig.2). For 7 RCT studies, SFR was 84.5% (1300 of 1538 patients) of Mini-PCNL and 83.8% (1275 of 1521 patients) of Standard-PCNL with no significant difference (RR=1.01, 95% CI 0.98–1.04, *P*=0.56; Fig.2). For 8 Non-RCT studies, SFR was 86.4% (610 of 706 patients) of Mini-PCNL and 84.1% (427 of 508 patients) of Standard-PCNL with no significant difference (RR=0.97, 95% CI 0.93–1.02, *P*=0.19; Fig.2).

### Operation time and subgroup analysis

**Fig.3:**
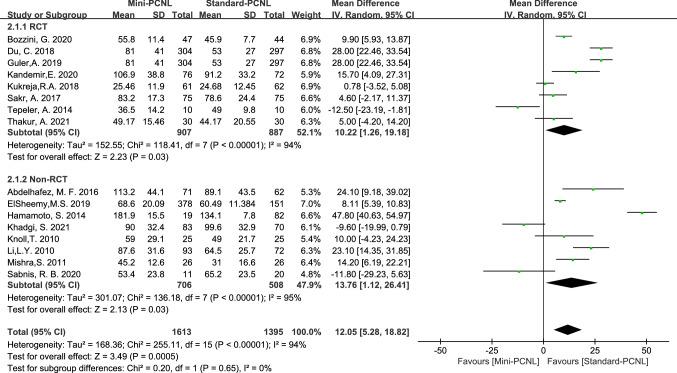
Forest plot for operative time

Operative time was reported in 16 studies with high heterogeneity across studies (*P*<0.00001, *I*^2^=94%), clinical consistency across studies after sensitivity analysis, and shorter operative time of Standard-PCNL using a random-effects model analysis (Mean Difference (MD) = 12.05, 95% CI 5.28–18.82, *P*=0.0005). In 8 RCT studies, Standard-PCNL was associated with shorter operative times using a random-effects model analysis (MD = 10.22, 95% CI 1.26–19.18, *P*=0.03; Fig.3); and there was the same conclusion reached in 8 Non-RCT studies (MD=13.76, 95% CI 1.12–26.41, *P*=0.03; Fig.3).

### Hospital stays and subgroup analysis

**Fig.4:**
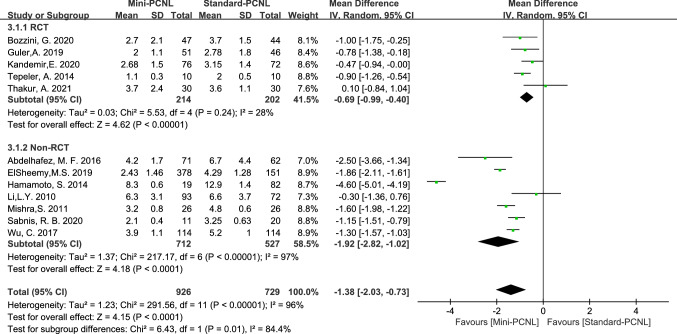
Forest plot for hospital stays

12 studies reported hospital stays, which was heterogeneous across studies (*P*<0.00001, *I*^2^=96%), clinical consistency across studies after sensitivity analysis, and shorter length of hospital stays with Mini-PCNL using random-effects model analysis (MD=–1.38, 95% CI –2.03 to –0.73, *P*<0.0001; Fig.4). In 5 RCT studies, Mini-PCNL had shorter hospital stay using random-effect model analysis (MD=–0.69, 95% CI –0.99 to –0.40, *P*<0.00001; Fig.4); and there was same conclusion in 7 Non-RCT studies (MD =–1.92, 95% CI –2.82 to –1.02, *P*<0.0001 Fig.4).

### Hemoglobin drop and subgroup analysis

**Fig.5:**
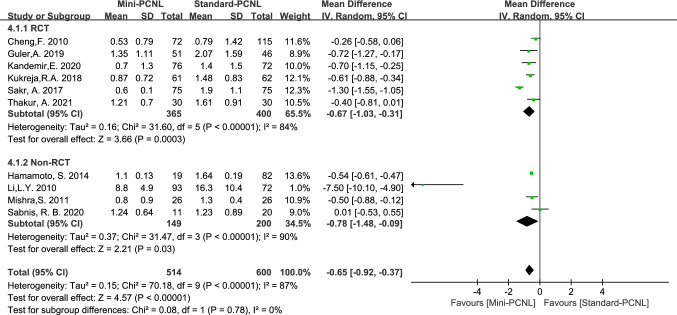
Forest plot for hemoglobin drop

10 studies reported hemoglobin drop, and less hemoglobin drop was found in Mini-PCNL using random-effects model (MD = –0.65, 95%CI –0.92 to –0.37; *P* < 0.00001; Fig. 5). Mini-PCNL showed less hemoglobin drop in 6 RCT studies analyzed with random-effects model (MD = –0.67, 95% CI –1.03 to –0.31, *P* = 0.0003; Fig. 5); and there was same conclusion in 4 Non-RCT studies (MD = –0.78, 95% CI –1.48 to −0.09, *P* = 0.03; Fig. 5).

### Blood transfusions and subgroup analysis

**Fig.6:**
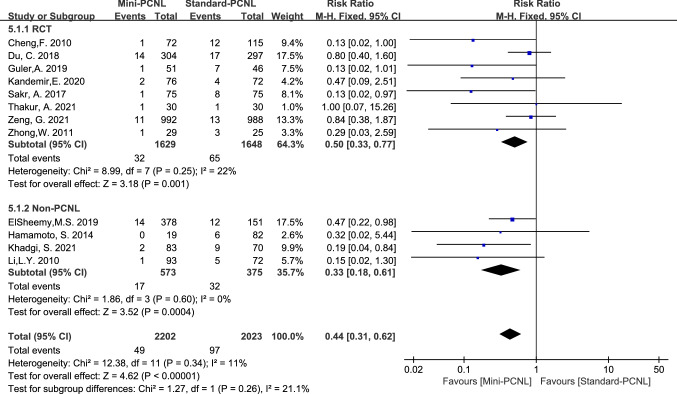
Forest plot for blood transfusions

12 studies reported blood transfusions, with good homogeneity among the studies (*P* = 0.34, *I*^2^ = 11%). Using the fixed-effect model analysis, meta-analysis results showed fewer blood transfusions in Mini-PCNL (RR = 0.44, 95% CI 0.31–0.62, *P* < 0.00001; Fig. 6). In 8 RCT studies, Mini-PCNL had fewer blood transfusions using a fixed-effects model analysis (RR = 0.50, 95% CI 0.33–0.77, *P* = 0.001; Fig. 6); and there was the same conclusion was reached in 4 Non-RCT studies (RR = 0.33, 95% CI 0.18–0.61, *P* = 0.0004; Fig. 6).

### Postoperative pain (VAS score) analysis

**Fig.7:**
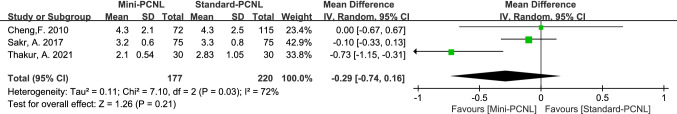
Forest plot for postoperative pain (VAS score)

3 studies reported postoperative pain (VAS score), which was heterogeneous across studies (*P* < 0.03, *I*^2^ = 72%), and there was clinical consistency across studies after sensitivity analysis, which was analyzed using a random-effects model, and there was no significant difference between the two groups (MD = –0.29, 95% CI –0.74 to 0.16, *P* = 0.21; Fig. 7).

### Tubeless PCNL rate analysis

**Fig.8:**
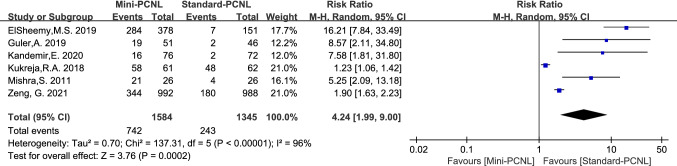
Forest plot for tubeless PCNL

6 studies reported tubeless PCNL, which was heterogeneous across studies (*P* < 0.00001, *I*^2^ = 96%), and there was clinical consistency across studies after sensitivity analysis, which was analyzed using a random-effects model. The report showed that Mini-PCNL has a higher tubeless rate (RR = 4.24, 95% CI 1.99–9.00, *P* = 0.0002; Fig. 8).

### Complications and subgroup analysis

**Fig.9:**
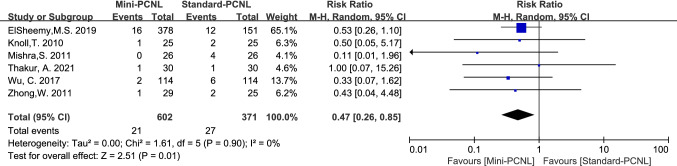
Forest plot for bleeding

**Fig.10:**
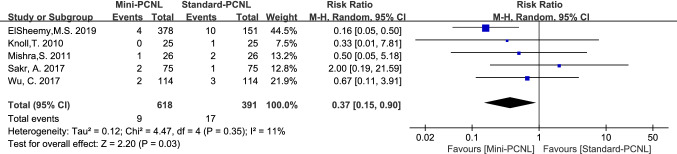
Forest plot for renal pelvis perforation

**Fig.11:**
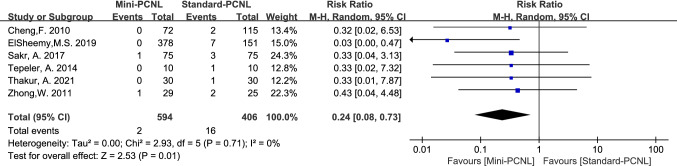
Forest plot for urine leakage

**Fig.12:**
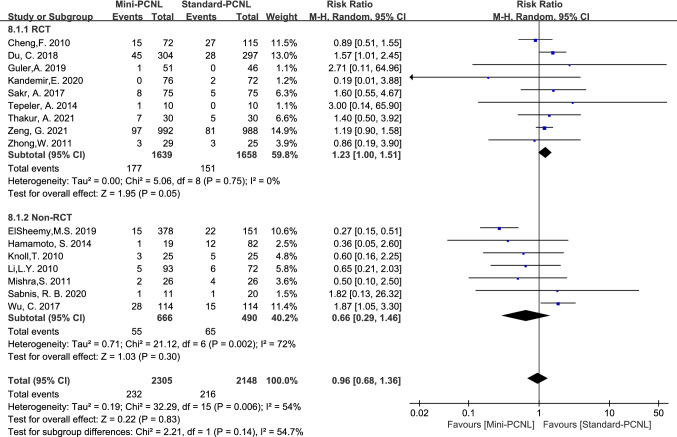
Forest plot for fever

Complications analyzed in this paper included: fever, bleeding, renal pelvis perforation, and urine leakage. Mini-PCNL is less likely to cause bleeding (RR = 0.47, 95%CI 0.26–0.85, *P* = 0.01; Fig. 9), renal pelvis perforation (RR = 0.37, 95%CI 0.15–0.90, *P* = 0.03; Fig. 10), and urine leakage (RR = 0.24, 95% CI 0.08–0.73, *P* = 0.01; Fig. 11). Standard-PCNL was clinically consistent across studies after a sensitivity analysis. However, there was no significant difference between Mini-PCNL and Standard-PCNL on fever (RR = 0.96, 95% CI 0.68–1.36, *P* = 0.83; Fig. 12).

### Publication bias

In this study, funnel plots were employed to assess publication bias. (Supplementary Table 4–14). The results were as follows: the funnel plot was symmetrical for blood transfusion, bleeding, and renal pelvis perforation; the funnel plot was basically symmetrical for SFR, operation time, hospital stay, hemoglobin drop, postoperative pain (VAS score), fever, urine leakage, and tubeless PCNL.

### Sensitivity analysis

The studies were removed in turn to investigate the effect of each study on the summary results. The pooled results did not show alterations when individual studies were excluded.

## Discussion

PCNL-based procedures, with the exception of a few patients with particularly big and/or intricate staghorn stones, are recommended preferred open surgery because to lower morbidity [27]. In recent years, Mini-PCNL has grown in popularity, and we analyzed the literature and discovered that it has a similar SFR to Standard-PCNL, but with shorter hospital stays, less hemoglobin drop, less blood transfusions, higher tubeless PCNL, and fewer complications, increased operative time.

SFR is one of the main indicators for surgeons to choose surgical approach. Lahme, S. et al. suggested that Standard-PCNL has a high stone-free rate, but it also has a high treatment morbidity rate [28]. However, in our study, we found that Mini-PCNL may have a similar SFR to Standard-PCNL in recent years. RCT literature study of subgroup analysis revealed two primary causes for the increased SFR of Mini-PCNL: 1. In terms of surface area, the nephroscope utilized in Standard-PCNL (20.8F) has a 150 percent increase over Mini-PCNL (12F). As a result, the distance between the tract and the nephroscope was larger with Mini-PCNL, allowing for improved visualization and fragment evacuation during the treatment [25]. 2. The presence of a large number of calculi (10.8 cm^2^) in the PCNL group in this series, as well as a lack of experience with flexible nephroscopy, may have contributed to a lower clearance rate than in other series [26]. For Non-RCT studies, we discovered that patients with many stones and a substantial stone burden > 2 cm^2^ had a significant difference in SFR, but patients with a single stone or a stone burden ≤ 2 cm^2^ had no significant difference in SFR [11]. We believe that, with the advancement of technology and equipment, Mini-PCNL might have a similar SFR to Standard-PCNL.

The analysis concluded that operating time was shorter in Standard-PCNL than in Mini-PCNL, whether in RCT or non-RCT studies. Because of the bigger sheath of Standard-PCNL and the clearance between the nephroscope and the channel, it is not necessary to break the stone into smaller fragments like Mini-PCNL, resulting in a shorter operative time.

We reviewed the literature in recent years and discovered that Mini-PCNL has a greater tubeless rate, which demonstrated superiority of Mini-PCNL. Mini-PCNL has a smaller wound bed, less bleeding, and less hemoglobin drop than Standard-PCNL, resulting in a higher tubeless rate, and potentially shorter hospital stays and less postoperative pain. Because the VAS score was used as the postoperative pain inclusion criterion in this study, there were only a few literatures eventually included, and the analysis results may be skewed as a result of the limited data extraction and quantity of literatures. Corroboration will require more high-quality literature items.

Finally, in the analysis of postoperative complications, Mini-PCNL was superior in terms of bleeding, perforation, and leakage due to its smaller sheath. In the analysis of fever, there was no significant difference between the two groups (Fig. 12). The main cause of postoperative fever in patients was believed to be bacterial endotoxin absorption produced by higher renal pelvis pressure in Mini-PCNL. However, some studies have shown that the Mini-PCNL nephroscope was at least 6.5 Fr smaller than the sheath (8.5/11.5 Fr ureteroscope in an 18 Fr sheath) [11]. The incidence of fever after Mini-PCNL was reduced as a result of this. The higher rate of fever after Standard-PCNL, on the other hand, could be due to the presence of infection calculi or a higher rate of complications: Perforation, leakage, hematoma, and obstruction of the pelvic–calyceal canal [11].

A number of enhancements should be made in the future. First, some articles have tiny sample sizes, which may necessitate larger sample sizes to confirm the articles' credibility. Second, different conclusions appear to be reached in RCT and non-RCT investigations; further research may be required for confirmation. Finally, despite applying the random-effects model to these elements, the study discovered considerable heterogeneity for some parameters, which may have an impact on the outcomes of our investigation. Despite these limitations, our meta-analysis offered high-quality evidence by updating the most recent data.

## Conclusion

According to our meta-analysis, Mini-PCNL is at least as effective and safe for the removal of renal calculi as Standard-PCNL with similar SFR. Furthermore, Mini-PCNL had a shorter hospital stay, less hemoglobin drop, less transfusion, greater postoperative tubeless, and fewer complications than Standard-PCNL.

## Supplementary Information

Below is the link to the electronic supplementary material.

Supplementary file1 (PDF 62 KB)

Supplementary file2 (PDF 43 KB)

Supplementary file3 (PDF 66 KB)

Supplementary file4 (PDF 63 KB)

Supplementary file5 (PDF 55 KB)

Supplementary file6 (PDF 52 KB)

Supplementary file7 (PDF 46 KB)

Supplementary file8 (PDF 57 KB)

Supplementary file9 (PDF 33 KB)

Supplementary file10 (PDF 41 KB)

Supplementary file11 (PDF 58 KB)

Supplementary file12 (PDF 35 KB)

Supplementary file13 (PDF 35 KB)

Supplementary file14 (PDF 35 KB)
