# Supplementary material for: Comparison of postoperative outcomes of mini percutaneous nephrolithotomy and standard percutaneous nephrolithotomy: a meta-analysis
Source: Urolithiasis. 2022 Aug 11;50(5):523–33. doi: 10.1007/s00240-022-01349-8 (PMC9467966; doi:10.1007/s00240-022-01349-8)
Supplement: Supplementary file 1 — Supplementary file1 (PDF 62 KB) [file 240_2022_1349_MOESM1_ESM.pdf]

Jadad Scale: Randomized Controlled Trial

Study	Was the study described as randomized?	Was the study described as double blind?	Was there a description of withdrawals and dropouts?	Total Score
Bozzini, G. 2020	2	0	1	3
Cheng,F. 2010	1	0	1	2
Du, C. 2018	1	0	1	2
Guler,A. 2019	2	0	1	3
Kandemir,E. 2020	1	0	1	2
Kukreja,R.A. 2018	2	0	1	3
Sakr, A. 2017	2	0	1	3
Tepeler, A. 2014	2	0	1	3
Thakur, A. 2021	1	0	1	2
Zeng, G. 2021	2	2	1	5
Zhong,W. 2011	1	0	1	2

Supplementary Table 1 Jadad Scale Randomized Controlled Trial
