# Supplementary material for: Comparison of postoperative outcomes of mini percutaneous nephrolithotomy and standard percutaneous nephrolithotomy: a meta-analysis
Source: Urolithiasis. 2022 Aug 11;50(5):523–33. doi: 10.1007/s00240-022-01349-8 (PMC9467966; doi:10.1007/s00240-022-01349-8)
Supplement: Supplementary file 2 — Supplementary file2 (PDF 43 KB) [file 240_2022_1349_MOESM2_ESM.pdf]

Table 1. Characteristics of included studies

study	Design	Procedures	Sample size	Age (year)	Sex (M/F)	side (R/L)	BMI, kg/m2	Stone Size, mm
Bozzini, G. 2020	RCT	Mini-PCNL	47	55.8	20/27	22/25		16.82
		Standard-PCNL	44	53.3	23/21	25/19		16.38
Cheng,F. 2010	RCT	Mini-PCNL	72	37.2	39/33	43/29		
		Standard-PCNL	115	39.6	63/52	67/48		
Du, C. 2018	RCT	Mini-PCNL	304	41.2±16.9	181/123	147/157		
		Standard-PCNL	297	44.5±18.7	179/118	151/146		
Guler,A. 2019	RCT	Mini-PCNL	51	46.9±13.7	29/22	29/22	28.5±5.6	38.7±13.1
		Standard-PCNL	46	47.4±13.9	23/23	25/21	29.6±5.9	42.8±22.5
Kandemir,E. 2020	RCT	Mini-PCNL	76	47.0±13.9	50/26	40/36	28.6±5.4	32.6±8.1
		Standard-PCNL	72	46.7±14.2	48/24	35/47	28.4±5.6	33.1±10.9
Kukreja,R.A. 2018	RCT	Mini-PCNL	61	41.95±13.53		33/28	27.1±5.87	20.6±3.47
		Standard-PCNL	62	40.3±14.2		30/32	25.54±3.58	21.5±3.53
Sakr, A. 2017	RCT	Mini-PCNL	75	43.8	40/35	51/36	28.4	27
		Standard-PCNL	75	40.2	52/23	33/48	27.8	26
Tepeler, A. 2014	RCT	Mini-PCNL	10	47.2	4/6		27.5	19.9
		Standard-PCNL	10	44.3	6/4		27.8	21.9
Thakur, A. 2021	RCT	Mini-PCNL	30	34.5±16.32	21/9		26.32±5.10	17.9±5
		Standard-PCNL	30	32.4±12.6	17/13		25±5.16	19.4±5.3
Zeng, G. 2021	RCT	Mini-PCNL	992	51	526/466	500/492	24.4	29
		Standard-PCNL	988	51	531/457	487/501	24.7	29
Zhong,W. 2011	RCT	Mini-PCNL	29	41	14/15			
		Standard-PCNL	25	38	11/14			
Abdelhafez, M. F. 2016	Non-RCT	Mini-PCNL	71	52	37/34	29/42	26.2	38.6
		Standard-PCNL	62	58	31/31	21/41	26.4	38.2
ElSheemy,M.S. 2019	Non-RCT	Mini-PCNL	378	37.08±12.62	137/241	206/172	27.2±2.22	
		Standard-PCNL	151	43.42±13.21	58/93	75/76	27.03±2.16	
Hamamoto, S. 2014	Non-RCT	Mini-PCNL	19	48.9	12/7	5/14	24.8	
		Standard-PCNL	82	53.2	66/16	22/60	24.6	
Khadgi, S. 2021	Non-RCT	Mini-PCNL	83	43.7±13.9	44/39	36/47	29±3.3	
		Standard-PCNL	70	51.9±9.7	32/38	21/41	34±6	

Knoll,T. 2010	Non-RCT	Mini-PCNL	25	52±11.6	16/9		27±3.5	18±3.3
		Standard-PCNL	25	48±15.5	17/8		29±5.6	22±4.25
Li,L.Y. 2010	Non-RCT	Mini-PCNL	93	51.5	56/37	48/45		28.6
		Standard-PCNL	72	49.2	43/29	31/41		30.4
Mishra,S. 2011	Non-RCT	Mini-PCNL	26	42.2±19.8	18/8	8/19	23.8±2.6	
		Standard-PCNL	26	48.2±16.8	18/8	10/18	22.6±2.7	
Sabnis, R. B. 2020	Non-RCT	Mini-PCNL	11	40.2±15.1	5/6			
		Standard-PCNL	20	49.2±11.5	16/4			
Wu, C. 2017	Non-RCT	Mini-PCNL	114	47.6±8.2	69/45	59/55	23.0±2.7	34±10
		Standard-PCNL	114	48.1±7.9	68/46	55/59	22.8±2.8	33±11
