# Supplementary material for: Comparison of postoperative outcomes of mini percutaneous nephrolithotomy and standard percutaneous nephrolithotomy: a meta-analysis
Source: Urolithiasis. 2022 Aug 11;50(5):523–33. doi: 10.1007/s00240-022-01349-8 (PMC9467966; doi:10.1007/s00240-022-01349-8)
Supplement: Supplementary file 3 — Supplementary file3 (PDF 66 KB) [file 240_2022_1349_MOESM3_ESM.pdf]

Newcastle-Ottawa Quality Assessment Scale: Cohort Studies

Study	Selection				Comparability	Outcome			
	Representativeness of the exposed cohort	Selection of the non exposed cohort	Ascertainment of exposure to implants	Demonstration that outcome of interest was not present at start of study	Comparability of cohorts on the basis of the design or analysis	Assessment of outcome	Was follow up long enough for outcomes to occur	Adequacy of follow up of cohorts	Total Score
ElSheemy,M.S. 2019	☆	/	☆	☆	☆	☆	☆	☆	7
Knoll,T. 2010	☆	/	☆	☆	☆	☆	☆	☆	7
Mishra,S. 2011	☆	/	☆	☆	☆	☆	☆	☆	7
Sabnis, R. B. 2020	☆	/	☆	☆	☆	☆	☆	☆	7
Wu,C. 2017	☆	/	☆	☆	☆	☆	☆	☆	7

Supplementary Table 3 Newcastle-Ottawa Quality Assessment Scale Cohort Studies
