# Supplementary figures and images for: Comparison of postoperative outcomes of mini percutaneous nephrolithotomy and standard percutaneous nephrolithotomy: a meta-analysis
Source: Urolithiasis. 2022 Aug 11;50(5):523–33. doi: 10.1007/s00240-022-01349-8 (PMC9467966; doi:10.1007/s00240-022-01349-8)

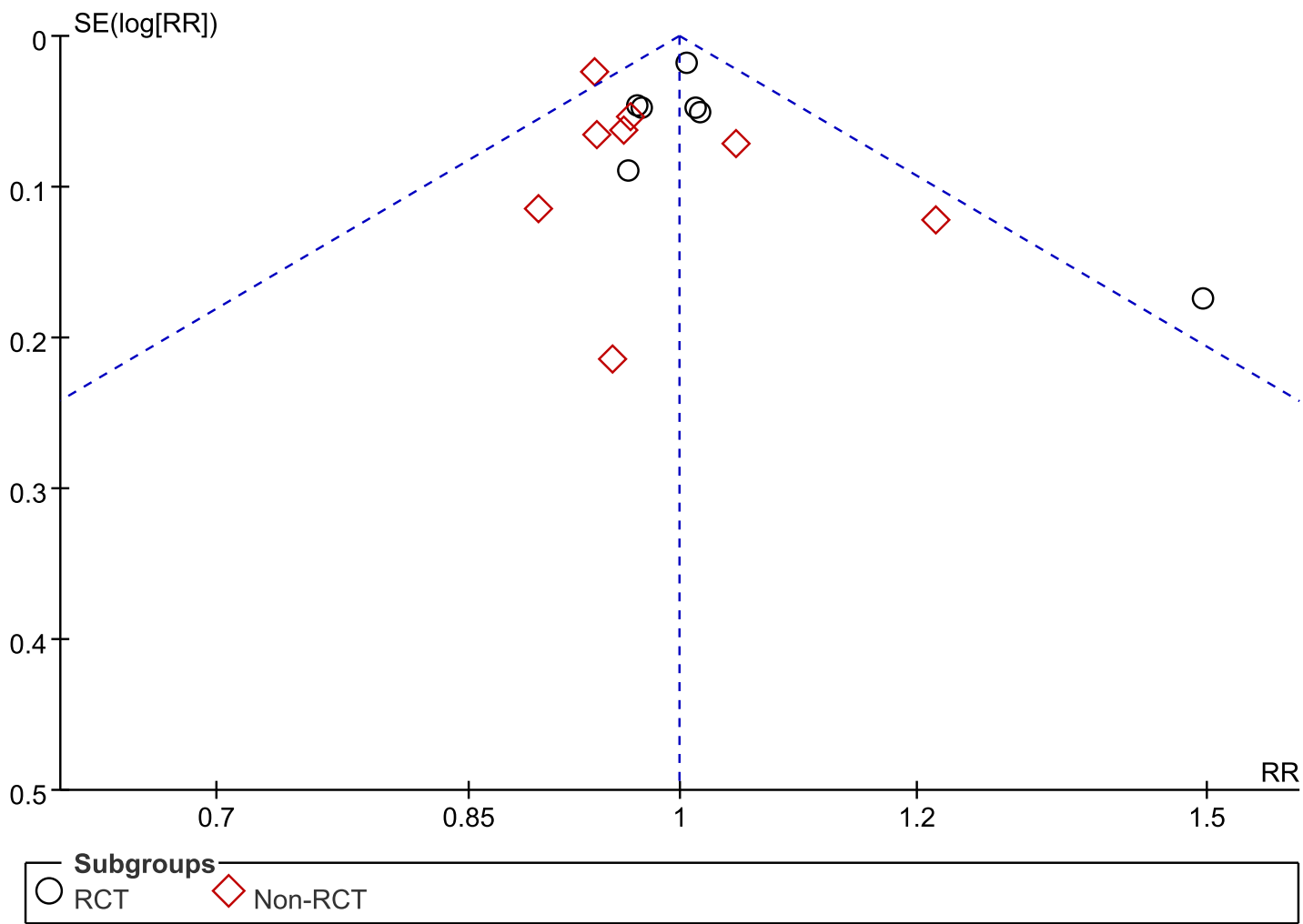

Supplementary Table 4 Funnel plot of SFR

Supplement: Supplementary file 4 — Supplementary file4 (PDF 63 KB) [file 240_2022_1349_MOESM4_ESM.pdf]

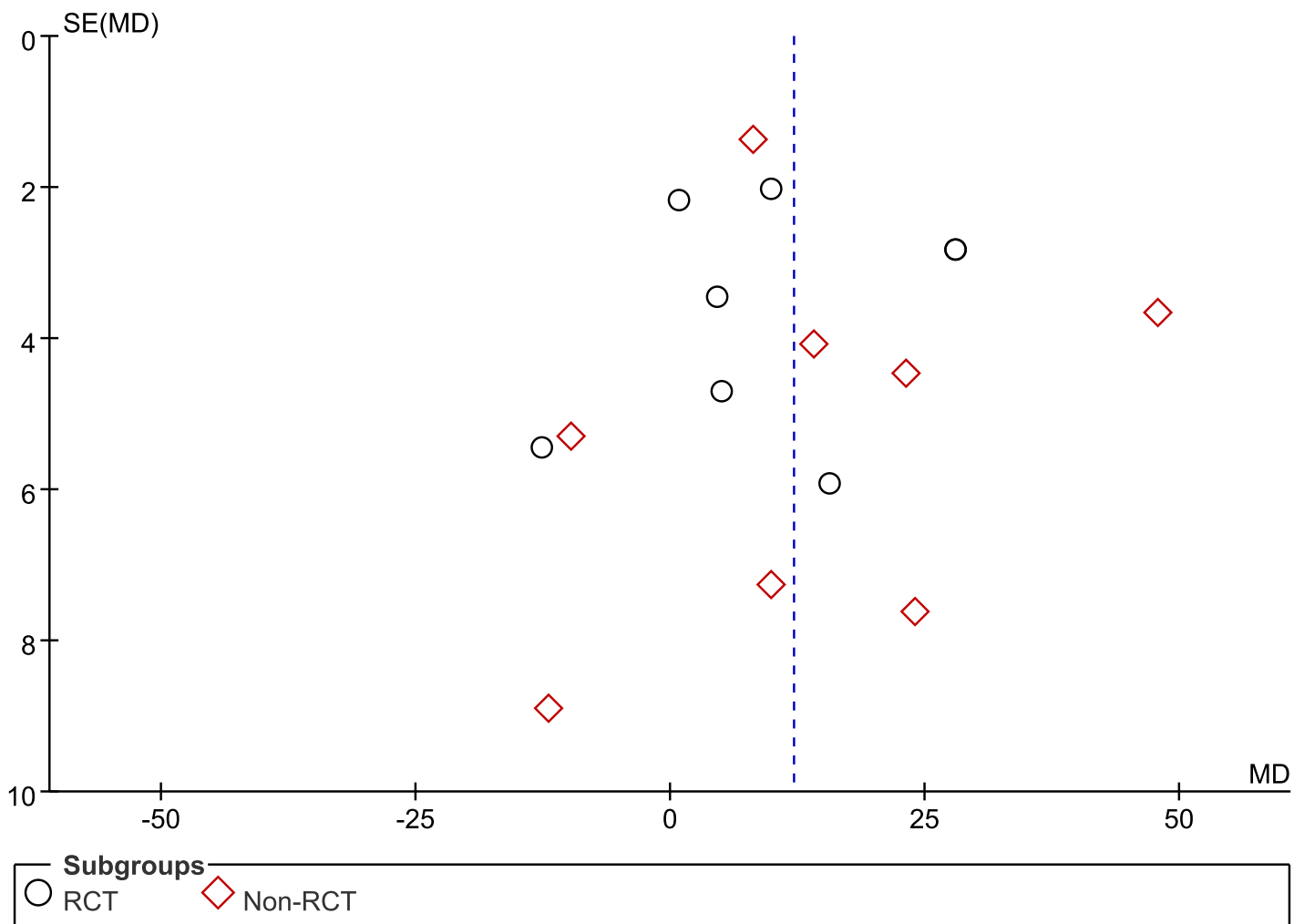

Supplementary Table 5 Funnel plot of Operative time

Supplement: Supplementary file 5 — Supplementary file5 (PDF 55 KB) [file 240_2022_1349_MOESM5_ESM.pdf]

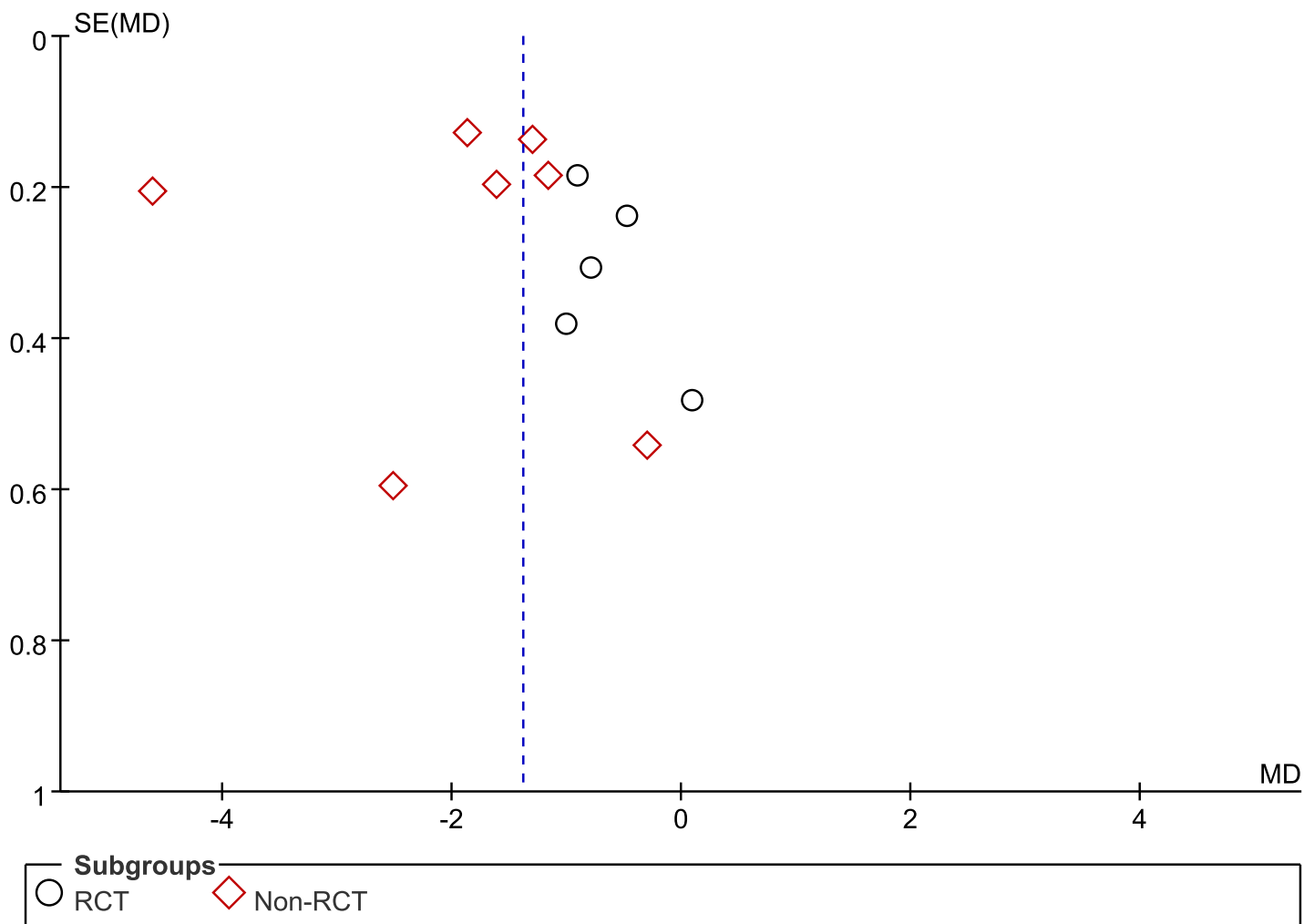

Supplementary Table 6 Funnel plot of Hospital stay

Supplement: Supplementary file 6 — Supplementary file6 (PDF 52 KB) [file 240_2022_1349_MOESM6_ESM.pdf]

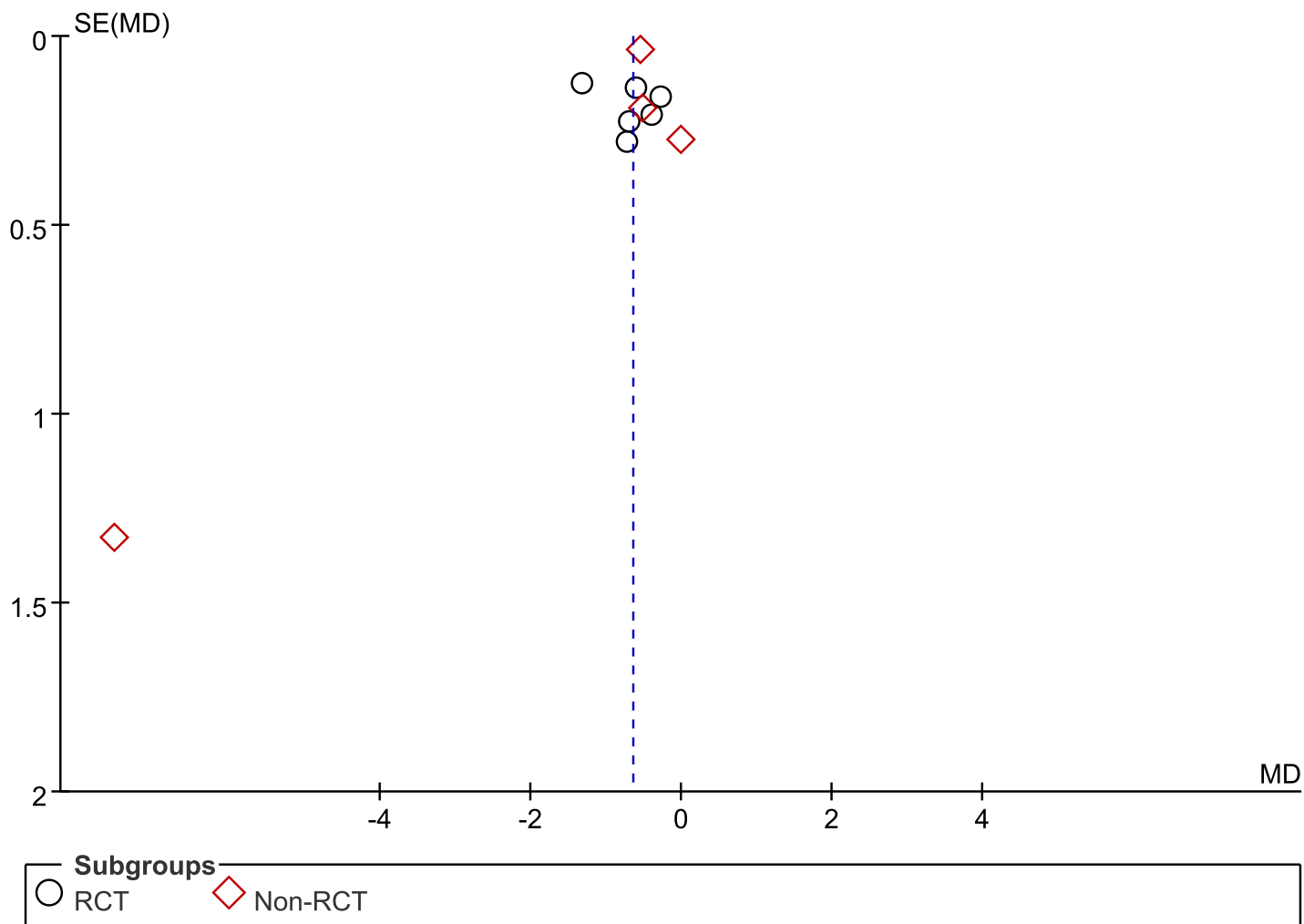

Supplementary Table 7 Funnel plot of Hemoglobin drop

Supplement: Supplementary file 7 — Supplementary file7 (PDF 46 KB) [file 240_2022_1349_MOESM7_ESM.pdf]

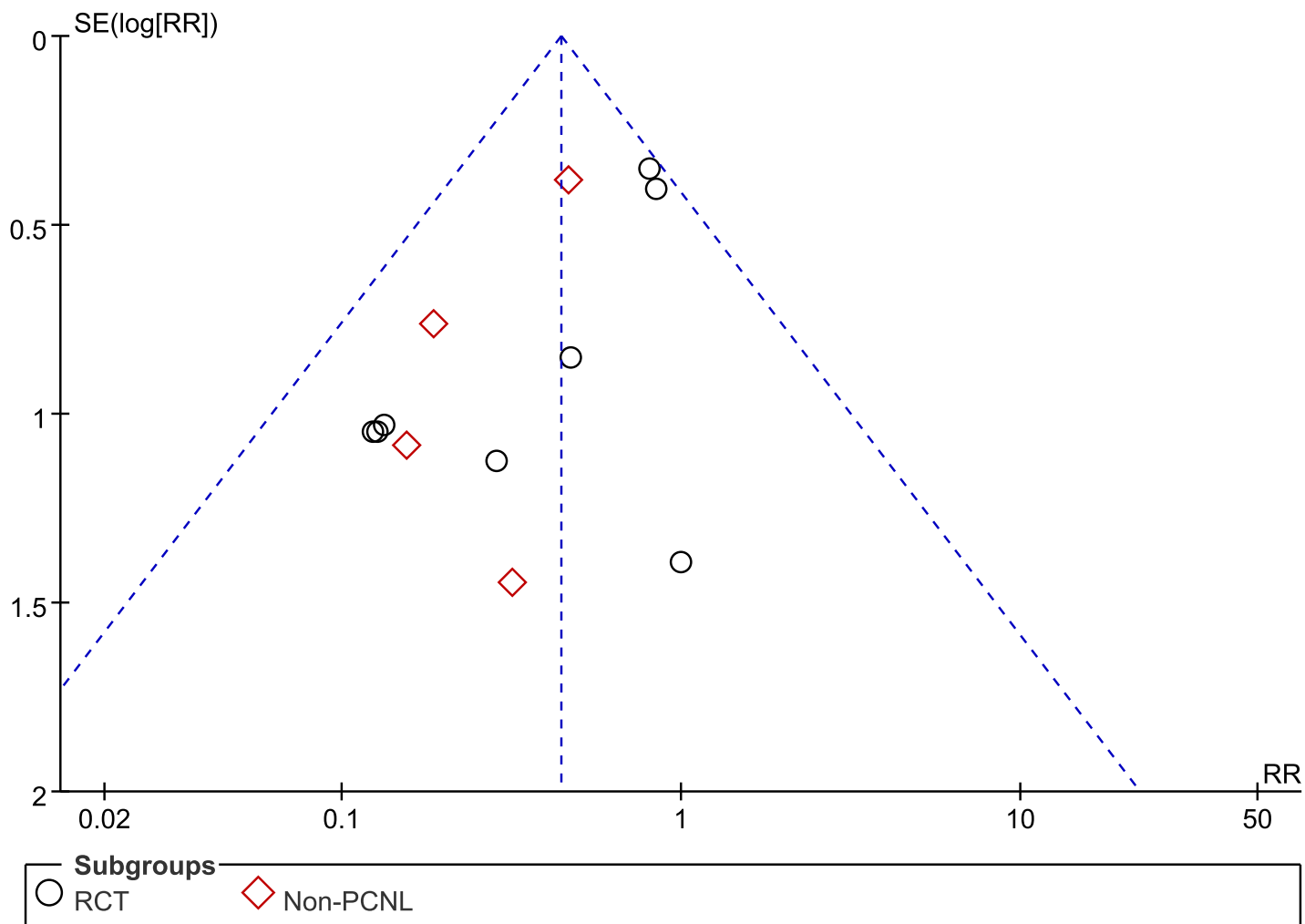

Supplementary Table 8 Funnel plot of transfusion

Supplement: Supplementary file 8 — Supplementary file8 (PDF 57 KB) [file 240_2022_1349_MOESM8_ESM.pdf]

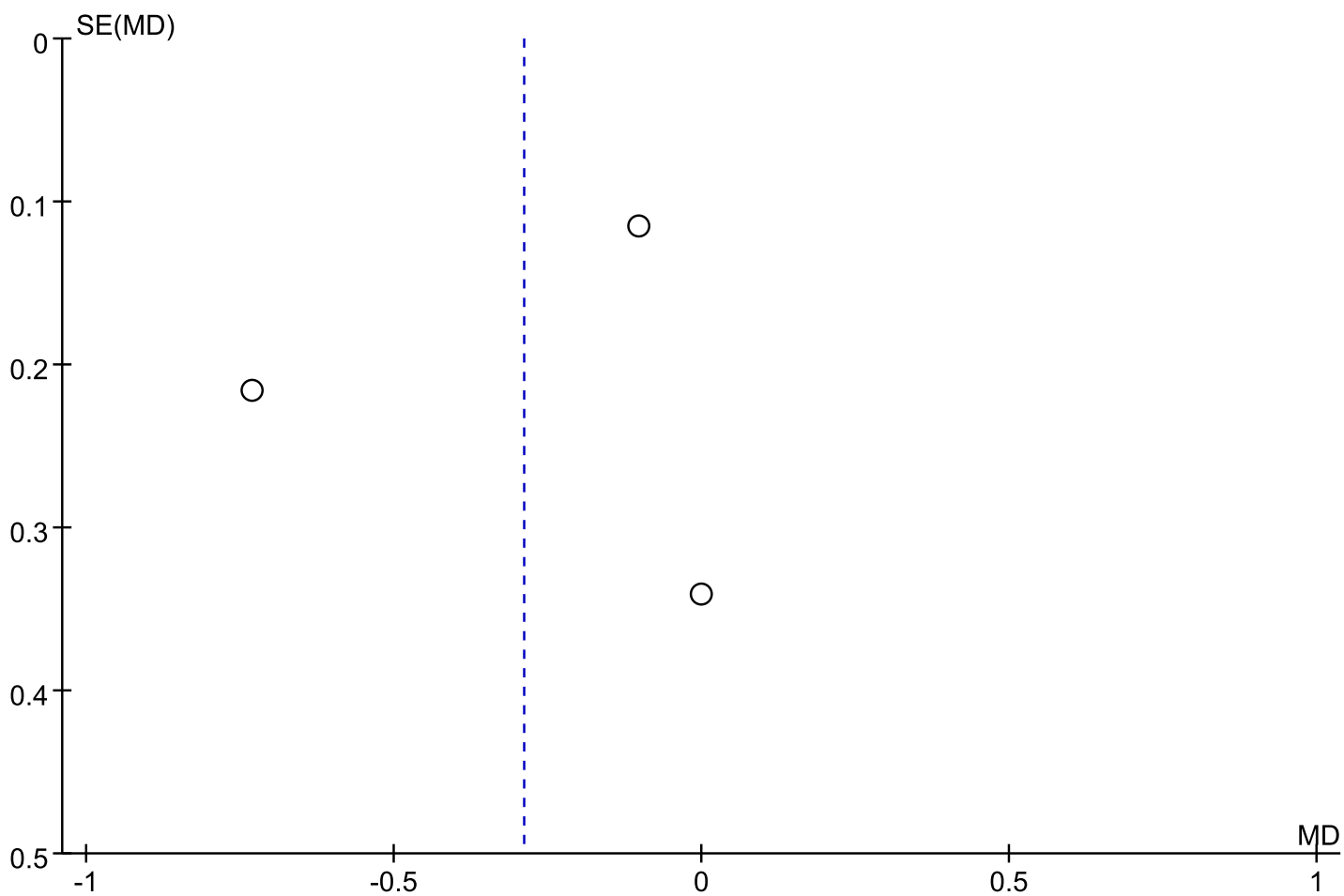

Supplementary Table 9 Funnel plot of VAS

Supplement: Supplementary file 9 — Supplementary file9 (PDF 33 KB) [file 240_2022_1349_MOESM9_ESM.pdf]

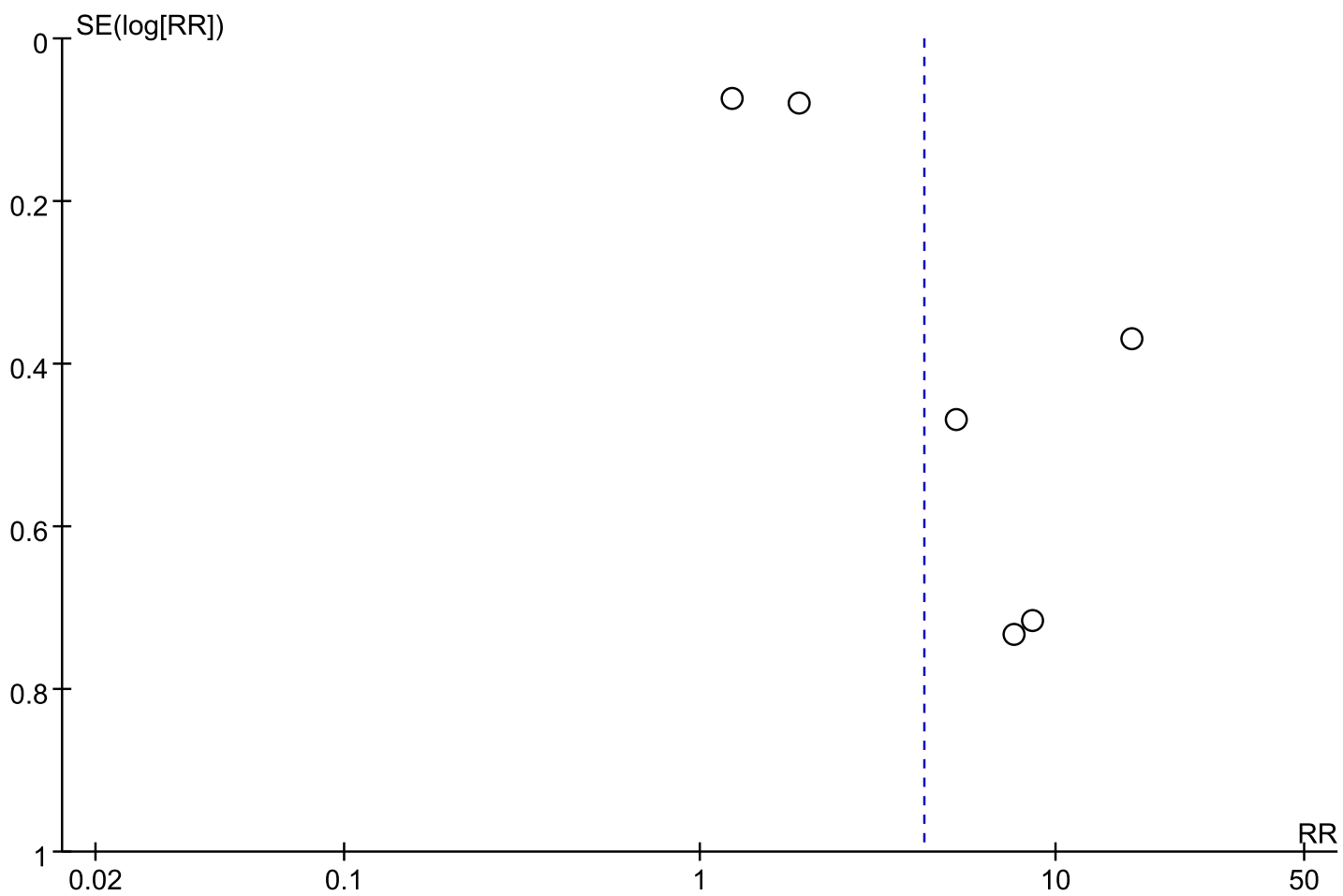

Supplementary Table 10 Funnel plot of tubless

Supplement: Supplementary file 10 — Supplementary file10 (PDF 41 KB) [file 240_2022_1349_MOESM10_ESM.pdf]

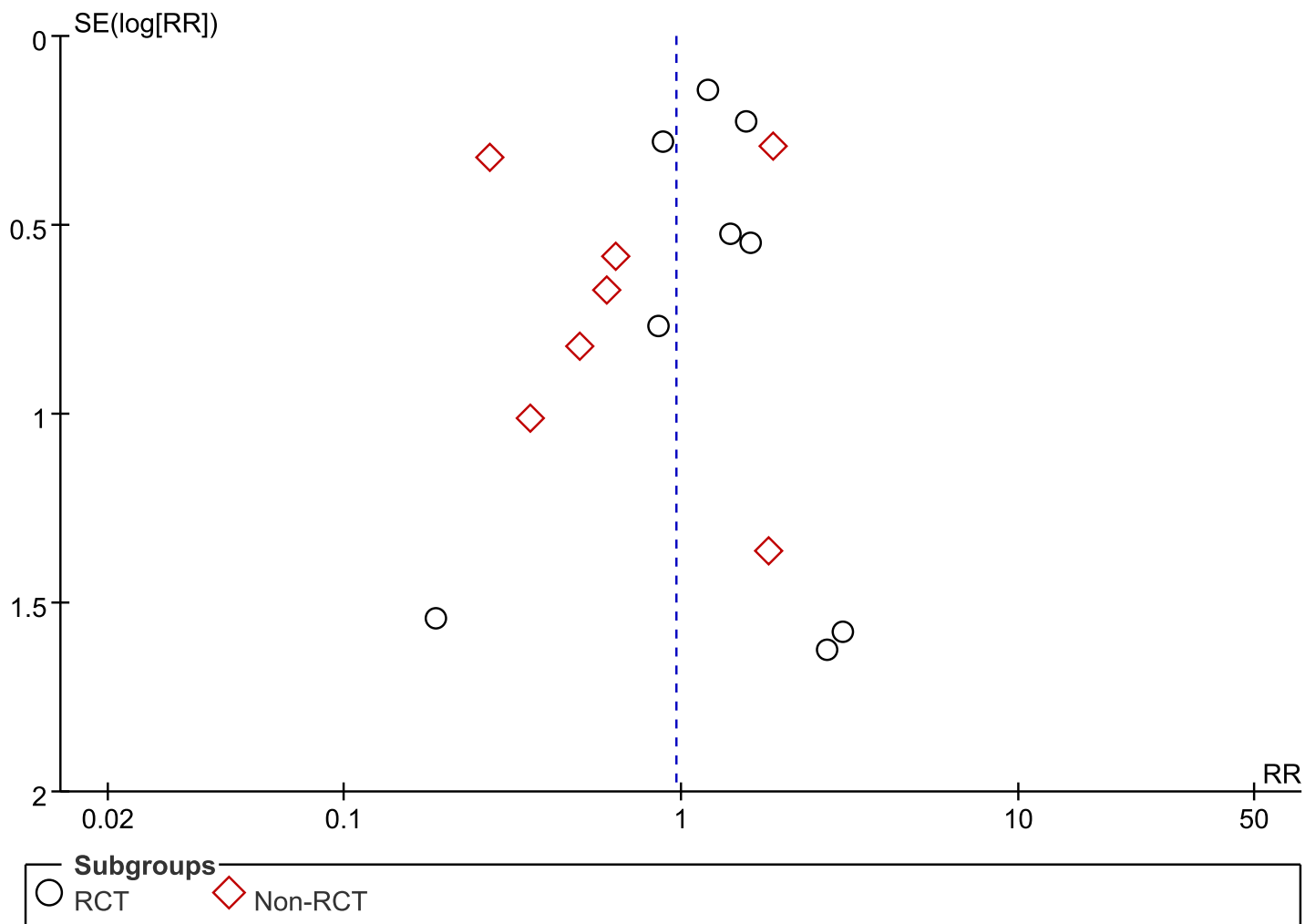

Supplementary Table 11 Funnel plot of fever

Supplement: Supplementary file 11 — Supplementary file11 (PDF 58 KB) [file 240_2022_1349_MOESM11_ESM.pdf]

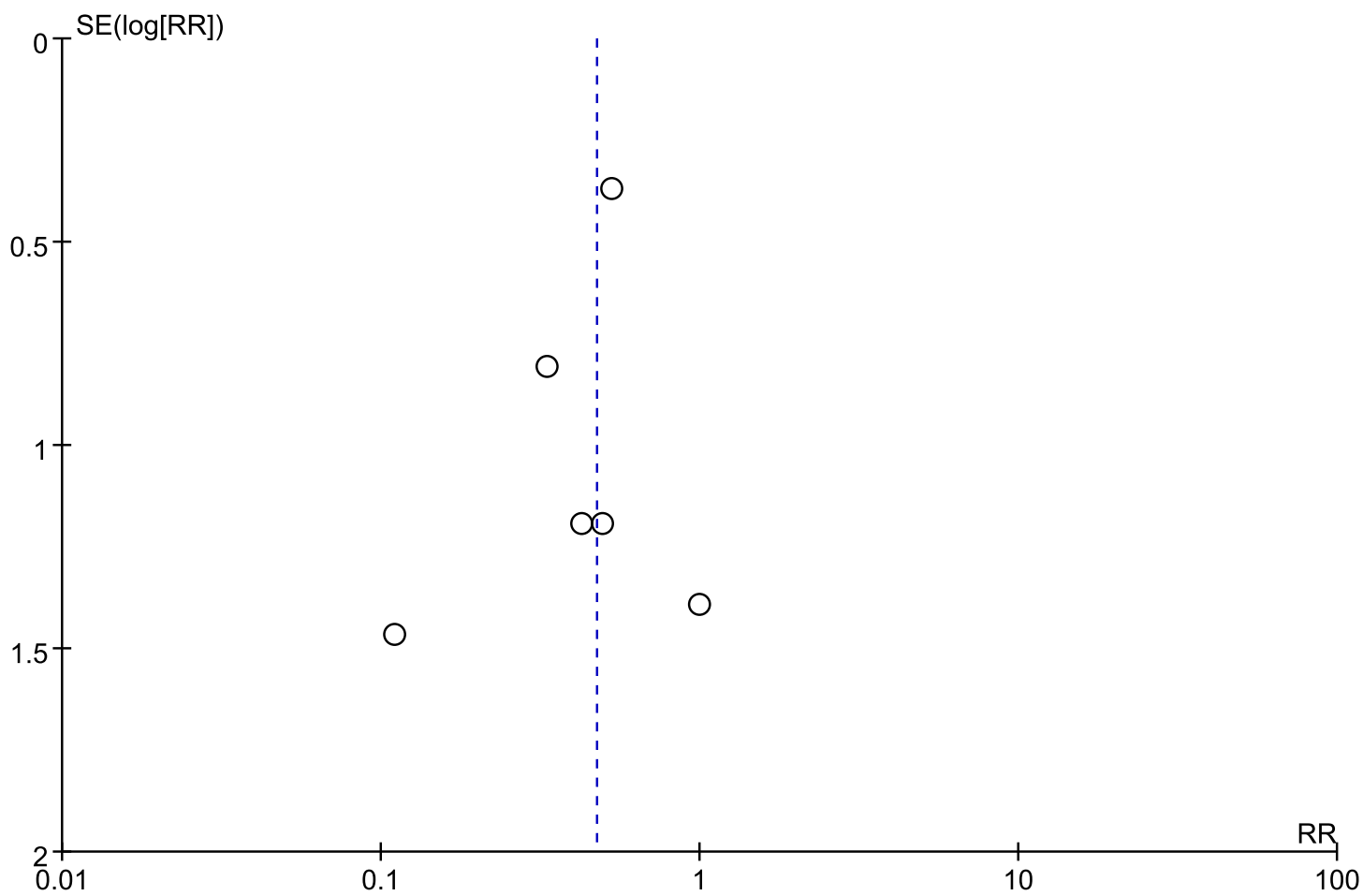

Supplementary Table 12 Funnel plot of Bleeding

Supplement: Supplementary file 12 — Supplementary file12 (PDF 35 KB) [file 240_2022_1349_MOESM12_ESM.pdf]

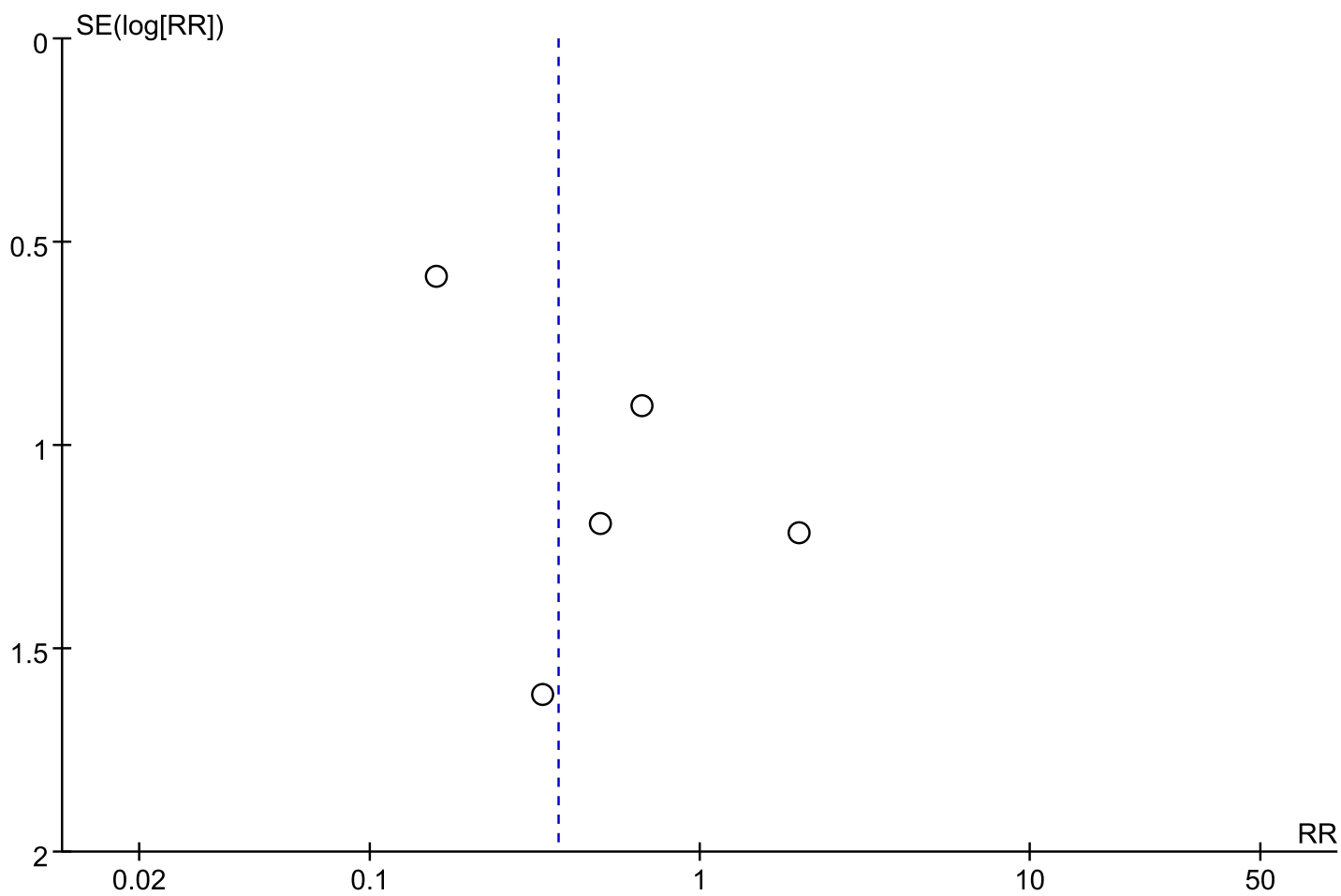

Supplementary Table 13 Funnel plot of Perforation

Supplement: Supplementary file 13 — Supplementary file13 (PDF 35 KB) [file 240_2022_1349_MOESM13_ESM.pdf]

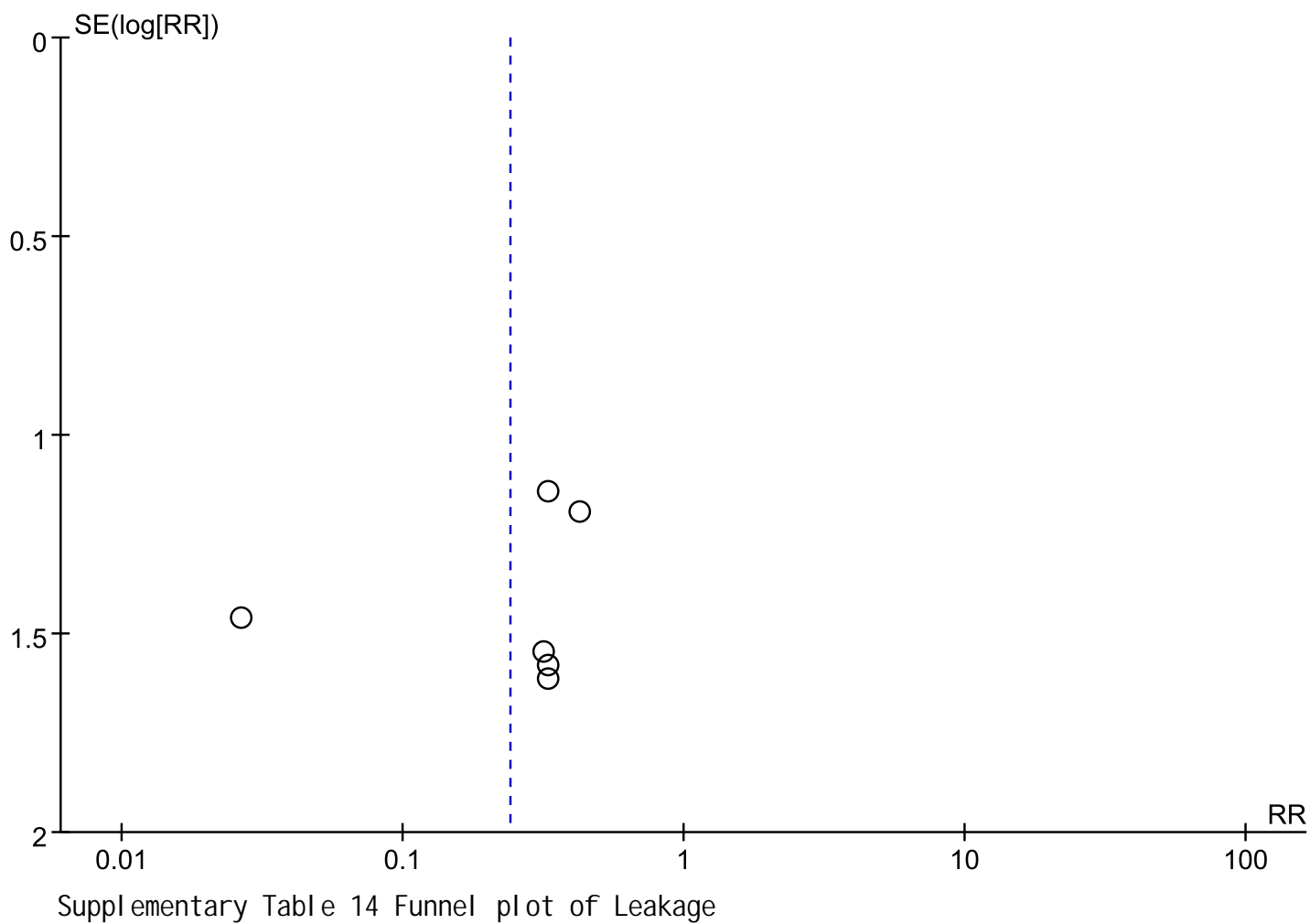

Supplement: Supplementary file 14 — Supplementary file14 (PDF 35 KB) [file 240_2022_1349_MOESM14_ESM.pdf]
